# The first case of transcatheter device closure of perimembranous ventricular septal defect in Nigeria: a case report

**DOI:** 10.11604/pamj.2023.44.88.36076

**Published:** 2023-02-15

**Authors:** Ogochukwu Jidechukwu Sokunbi, Bassey Olumide Udom, Naveen Kuzhippil Sreedhar, Michael Olutoyin Sanusi, Rajasekaran Premsekar

**Affiliations:** 1 Department of Paediatrics, College of Medicine, University of Lagos, Lagos University Teaching Hospital Idi-Araba, Lagos, Nigeria,; 2 Babcock/Tristate Heart and Vascular Centre, Babcock University Teaching Hospital, Ilishan Remo, Ogun State, Nigeria,; 3 Dr. Kamakshi Memorial Hospital, Chennai, India

**Keywords:** Transcatheter closure, ventricular septal defect, interventional paediatric cardiology, Nigeria, case report

## Abstract

Ventricular septal defect (VSD) is the most common congenital cardiac anomaly with a prevalence of 1.17 per 1000 live births. Haemodynamically significant VSDs require closure either surgical or transcatheter. We report a case of transcatheter device closure of a moderate-sized perimembranous ventricular septal defect (PmVSD), the first of its kind in Nigeria. The procedure was performed on a 23-month-old female weighing 10 kg who had presented with a history of frequent pneumonia and poor weight gain and signs of heart failure. The procedure was uncomplicated, and she was discharged 24 hours after the intervention. She had been followed-up two years post-procedure without complications and she had achieved appreciable weight gain. This non-surgical option was effective in this patient and provided the advantage of limited hospitalization, accelerated recovery, and intervention without the need for blood products. Such interventions should be scaled up in Nigeria and other sub-Saharan African countries.

## Introduction

Ventricular septal defect (VSD) is the most common congenital cardiac anomaly and isolated VSD accounts for 20% of all congenital heart defects with a prevalence of 1.17 per 1000 live births [1]. The prevalence of isolated VSD in Nigeria is like global figures with previous reports from two large centres documented as 13.9% and 18.6% respectively [2,3].

Ventricular septal defects are categorised based on their location; with 80% of the defects located in the membranous septum and termed “perimembranous”. Others include muscular, inlet and outlet or supra cristal VSD. Commonly, the defect is sized by comparing it to the diameter of the aortic valve annulus and is considered small when the ratio is less than 25%, moderate when between 25-75% and large when the defect exceeds 75% of the diameter of the aortic annulus. Moderate to large-sized ventricular septal defects are frequently complicated by congestive cardiac failure and pulmonary hypertension while small VSDs have been implicated as predisposing factors for infective endocarditis and worsening of tricuspid valve regurgitation and aortic regurgitation when VSDs are restricted by tricuspid valve septal aneurysm and aortic valve cusp prolapse respectively [4].

Surgical closure is the choice of management in VSDs associated with prolapse of the aortic valve cusp, aortic valve regurgitation or anomalous muscle bundle obstructing the right ventricular outflow tract. In the absence of these associations, transcatheter closure of perimembranous VSD (PmVSD) lends itself as an effective and safe alternative for interrupting interventricular shunts without a scar, significantly reduced pain, shorter hospital stay and cheaper cost when compared to the traditional open-heart surgery [5] with the added advantage of a significant reduction in mortality and complications [6]. While transcatheter device closure of patent ductus arteriosus and atrial septal defects have been reported previously in Nigeria [7,8]; to the best of our knowledge this is the first report of transcatheter closure of a PmVSD in the country.

## Patient and observation

**Patient information:** a 23-month-old female child had been diagnosed to have a moderate-sized PmVSD at four months of age.

**Clinical findings:** she presented with fast, laboured breathing and poor growth and a history of three previous episodes of chest infections. She had also been delivered at a birth weight of 1.05 kg on account of a twin-twin transfusion.

**Timeline of the current episode:** she had presented a year earlier at 11 months of age for pre-surgical evaluation for a moderate-sized PmVSD and had been receiving oral diuretics since diagnosis. Dietary counsel was given, medications optimized, and parents were counselled for early closure of the VSD. She presented for the procedure a year after, and parents were counselled regarding the benefits and risks of transcatheter closure as compared to the surgical option and the family opted for the former. A written and signed informed consent was obtained.

**Diagnostic assessment:** physical examination revealed no evidence of dysmorphism, no obvious respiratory distress, cyanosis or peripheral oedema. She weighed 10.0 kg which was 85% of her expected weight. The pulse rate was 116 bpm and pulses were normal volume, regular, synchronous, and well felt in all peripheries. Her precordium was active with the apex beat localised to the fourth left intercostal space lateral to the midclavicular line. Blood pressure was 100/49mmHg (90^th^ centile for both systolic and diastolic pressures) and first and second heart sounds were normal with a grade 3/6 pan systolic murmur at the mid left sternal border. Oxygen saturation by pulse oximetry was 98% in room air, respiratory rate was 48 per minute and breath sounds were vesicular. She had a soft, smooth, non-tender hepatomegaly, 4cm below the right costal margin and no other abdominal organ was palpably enlarged.

**Figure 1:**
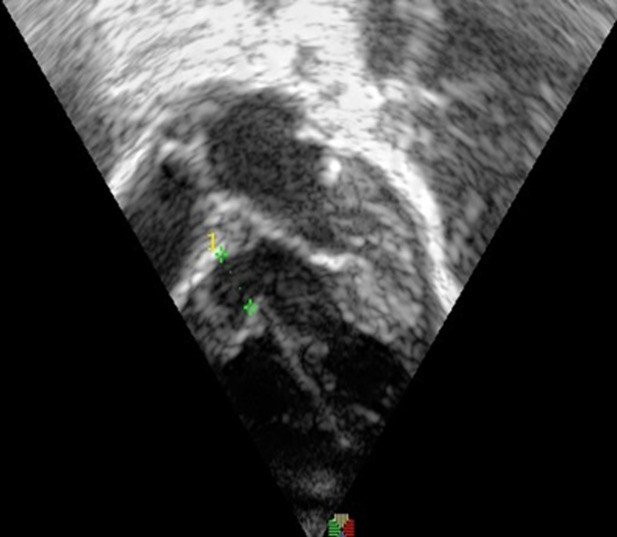
pre-procedure echocardiogram

**Diagnosis:** electrocardiography revealed sinus rhythm with a heart rate of 112 bpm, QRS axis of +300, predominant left ventricular forces and an incomplete right bundle branch block. Echocardiography showed a 7mm perimembranous ventricular septal defect partially restricted by the tricuspid valve septal leaflet aneurysm, shunting left to right (Figure 1). The peak interventricular pressure gradient was 80mmHg and the left atrium, left ventricle and pulmonary arteries were dilated with good biventricular function.

**Figure 2:**
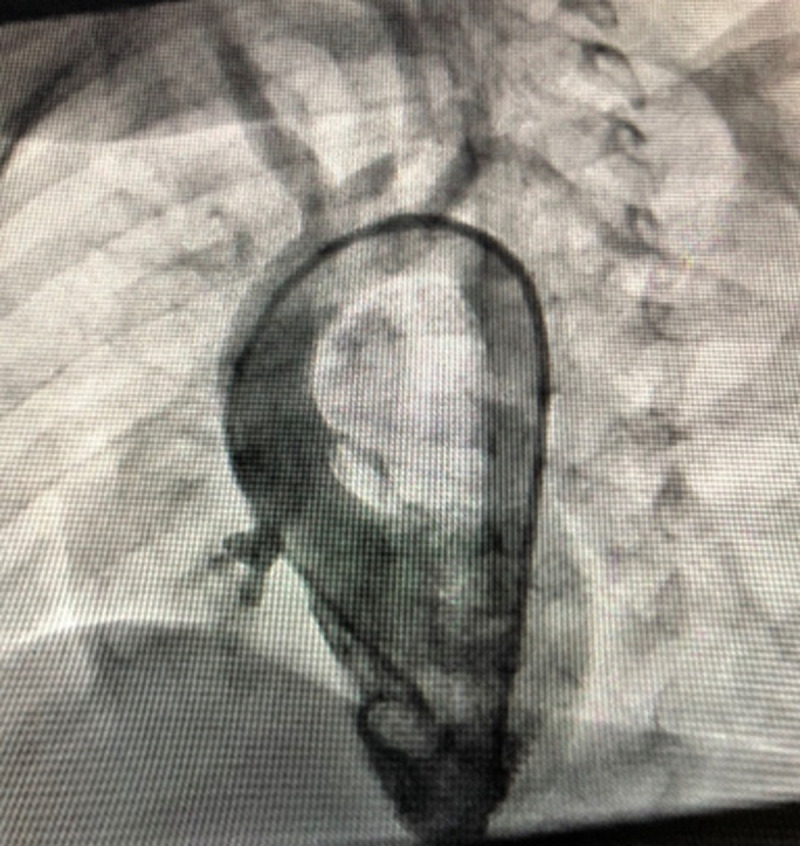
left ventriculogram

**Figure 3:**
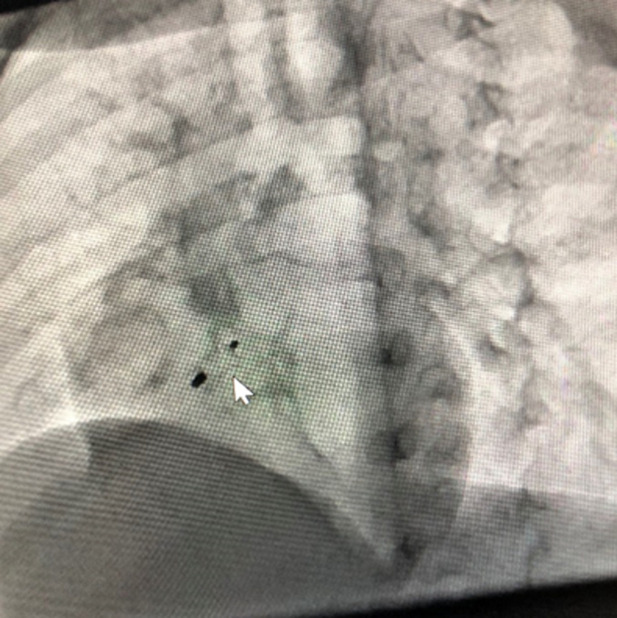
ventricular septal defect device post-release

**Therapeutic interventions:** the child underwent successful transcatheter VSD device closure with a 0608 Lifetech CeraTM duct occluder (Lifetech Scientific Corporation, Shenzhen, China) under general anaesthesia using a laryngeal mask airway. Both arterial and venous femoral accesses were obtained, and 100 units/kg of intravenous heparin was administered. The intravenous antibiotic was administered as per the institution's protocol. Pre-procedure transthoracic echocardiography (TTE) showed a PmVSD measuring 7.0mm and was restricted to 4.0mm by the tricuspid septal leaflet aneurysm. A left ventriculogram done in left anterior oblique (LAO) 400 cranial 200 view using a 5Fr pigtail catheter confirmed the echocardiography findings (Figure 2). The VSD was crossed retrograde from the aorta with a 5F Judkins Right catheter and 0.035” 260cm Terumo wire combination and the wire manoeuvred into the superior vena cava and then the innominate vein. It was then snared using a 4F 10mm LifetechTM snare catheter (Lifetech Scientific Corporation, Shenzhen, China) and exteriorised from the right femoral vein forming an arteriovenous (AV) loop. The femoral venous short sheath was exchanged for a 6F Steer Ease introducer sheath (Lifetech Scientific Corporation, Shenzhen, China) which was passed antegrade over the venous limb of the AV loop and the sheath tip placed in the left ventricle. The duct occluder device was prepared, loaded in the delivery sheath, and deployed across the PmVSD under fluoroscopic and TTE guidance and released (Figure 3). The procedure time was 77 minutes and the fluoroscopy time was 24.3 minutes.

**Figure 4:**
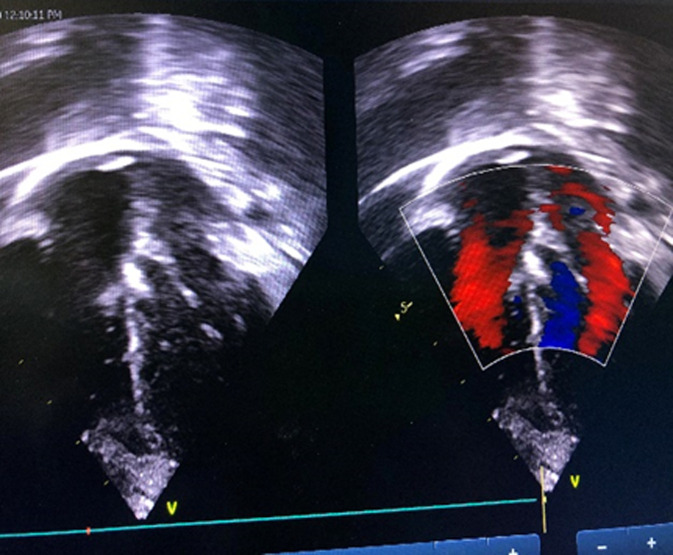
post-procedure echocardiogram

**Follow-up and outcome of interventions:** there were no procedural complications. Post-deployment TTE showed the device in a stable position with no residual shunt and no aortic valve regurgitation (Figure 4). Post-procedure electrocardiography showed normal sinus rhythm. The child was discharged 24 hours after the procedure in good health. Oral Aspirin 5mg/kg was prescribed for 6 months. By the third month following the procedure, activity had significantly improved, and she had gained 1.0 kg. She has been seen severally on follow-up; the last being two years post-intervention and has no complications.

**Patient perspective:** “we are happy that our child can now live a normal life after this procedure”. The parents expressed profound gratitude to the entire health team and were grateful to have had the benefit of state-of-the-art cardiovascular care in the country.

**Informed consent:** written and informed signed consent to publish de-identified data of the child was obtained from both parents.

## Discussion

The ventricular septum is formed by the sixth week of gestation because of a complex fusion of components from the atrioventricular cushions, the bulboventricular flange and the outflow tract ridges. Cell death after completion of septal formation is implicated in the formation of muscular defects while transient interruption of blood supply is attributed to failed fusion resulting in perimembranous defects [9]. While spontaneous closure occurs in 40 to 60% of patients [10], these are mainly muscular defects compared to those in the membranous septum [11]. A clinically symptomatic shunt with left heart dilatation and a systemic-to-pulmonary shunt ratio greater than 1.5 warrants closure. Surgical closure of VSD has been the traditional method but is not without complications [12].

Transcatheter management of VSD has several advantages including limited hospitalisation, accelerated recovery, little or no need for blood products as well as reduced risk of rhythm disturbances. Transcatheter closure of VSD has been reported as safe and effective in up to 99% of patients especially when patient and device selection is carefully done [13]. The morphology, size of VSD and weight of the patient are also factors that must be considered for the suitability of transcatheter treatment with reduced risk of complications.

Perimembranous ventricular septal defect device closure using duct occluders has been reported previously [14] and some of the advantages include a lower risk of complete heart block due to the use of smaller delivery sheaths, lower profile, and ease of implantation of the device which may reduce the trauma, clamp force, and radial stress to the ventricular septum [15]. Duct occluders are also more economical compared to standard VSD occluders and hence are favourable in resource-limited settings such as ours. Some other advantages of the use of duct occluders for PmVSD closure include the morphological similarity between peri-membranous defects with aneurysms and patent ductus arteriosus as well as the absence of a disc on the right ventricular aspect limiting the risk of the complete atrioventricular block as a complication of the procedure [16].

Transcatheter closure of VSD is particularly useful for postoperative residual shunts and post-myocardial infarction lesions where cardiopulmonary bypass constitutes an added risk [17]. Despite its advantages, not all VSDs are amenable to device closures such as inlet VSDs, co-existing significant aortic regurgitation and other concomitant lesions requiring surgical correction. However, with improvement in technique and availability of more recent configurations of devices, some VSDs which were previously contraindicated are infrequently reported as being successfully closed using the transcatheter approach [18].

Transcatheter VSD closure is however not without its complications. These include device embolization, aortic insufficiency, tricuspid insufficiency, bundle branch block, varying degrees of atrioventricular block, device-related arrhythmia, haemolysis, residual shunts and vascular complications [19]. Interventional treatment for VSD can be further improved by careful case selection, improved technology, and enhanced operator skill to reduce the incidence of complications.

The cost and availability of occluders still remain a major challenge in resource-limited settings like ours. With increasing reports of such interventional procedures being carried out in the country, it is hoped that it will help relax the policies of the government as well as ease the channels of distribution by the device manufacturers. This will ensure widespread local availability of occluders so that these procedures become routine. The establishment of sustainable insurance schemes to cover the cost of cardiac care is still a work in progress in Nigeria. The rapid turnover offered by transcatheter management of amenable cardiac defects enables care for more patients in a shorter duration, utilizing fewer consumables, intensive care unit resources and blood products. These features while making the procedures cost-effective in the long run for the cardiac centres, may also encourage the inclusion of such procedures under insurance schemes.

Cardiac surgery is yet to become commonplace in Nigeria as centres are still few and far between. This is the case in most sub-Saharan countries most of which can boast of a few centres which perform “walk-in” cardiac surgery and interventions. Even with a fully functional paediatric cardiothoracic surgical unit, resource-constrained environments where the majority of patients rely on out-of-pocket payments for healthcare will benefit from an alternate option for VSD closure which is not only safe and effective but also cheaper with limited hospitalisation. It is necessary to stress the importance of paediatric cardiac care as a multi-disciplinary team effort and that all complex interventional procedures like PmVSD device closures be undertaken with adequate surgical backup in the best interest of the child.

## Conclusion

We report the first case of transcatheter device closure of the perimembranous ventricular septal defect in Nigeria which was effective with no complications. Such interventions should be scaled up in Nigeria and other sub-Saharan African countries.
